# Domestic Medicine in South Africa

**Published:** 1901-11-23

**Authors:** 


### DOMESTIC MEDICINE IN SOUTH AFRICA.

THERE are probably few English families who have not, before now, read with something like a shock of horror the accounts of South African domestic medicine contained in the volume of reports issued last week by the War Office; or in which the loss of life among young children, for which this domestic medicine has been responsible, has not given rise to feelings of indignation, however much they may have been tempered by compassion for the ignorance of the people chiefly concerned. There is, indeed, something grotesque as well as horrible in the prosaic descriptions, written hastily and in few words by overworked doctors, of the bye-influences to which their patients have been subjected; in the descriptions, for example, of the children coated over with arsenical green paint, "all but their faces;" of the children so covered with accumulated dirt that they were as dark as Kaffirs, and that their skins could only be discovered after scraping; of the children to whom had been administered a compound containing ether, opium, tartar emetic, ginger, and other undisclosed ingredients, and who were "still unconscious" when the letter was ready for despatch; of the girl with renal dropsy who was enveloped from her waist downwards in horse-dung, in order to "take down the swelling from her face;" of the children to whom preparations of goat's dung and of horse dung, variously cooked in oil or in water, were administered either as cooling drinks or as healing agencies; of the children whom water, even in the form of poultices to their chests, was forbidden to approach, lest it should be productive of fatal consequences. The whole story is inexpressibly repulsive; but it owes its chief repulsiveness to the manner in which it exhibits the filthiness of Boer life. The knowledge just gained of the mental attitude of the Boer women towards water (it is curious that throughout the reports there is no mention of any corresponding antipathy to soap, which may possibly be an unknown commodity) may allow charitable people to place a new and better interpretation on what Mr. Swinburne describes as "the hellish atrocities of the Boer women," who early in the war "stood between Englishwomen, with their dying babies in their arms, and the water, a drop of which might have saved their lives on the horrible journey by rail, at the end of which they were dead of thirst." Miss Hobhouse and her like will perhaps tell us that the Boer women were fearful lest English mothers, in their blindness, might imperil the safety of their children by washing them, and were only anxious to prevent recourse being had to so objectionable and perilous a practice.

But, after all, if we set aside the filthiness alike of the "remedies" employed and of the persons employing them, or regard this filthiness as a mere national characteristic in which we, as a people, can scarcely be said to share, are we not in danger of being a little too self-righteous in our condemnation of the domestic medicine to which we have referred? Are there not great boasters among us? What is the real difference between a Boer mother who withholds the milk which the doctor has ordered for her baby, and gives it sardines instead, and the thousands of English mothers who swell the roll of our own infantile mortality by giving as food to the baby "whatever we have ourselves." What is the real difference between the Boer mother who administers the mixture to which reference is made above, and the English mother in whose nursery cupboard can be found bottles of soothing syrups and teething mixtures? By what distinguishing characteristic may we separate the anti-vaccinist, or, for the matter of that, the "anti" anything else, from his Boer sister and his gallant brother? More years ago than we like to remember, there was a little book called "Evenings at Home," by Dr. Aikin and Mrs. Barbauld, the delight at once of children and of children of a larger growth. Among other narratives, it contained an account, disguised under the form of a traveller's description of the manners and customs of certain savage islanders, of various toilet and other practices which were at that time current among the civilised contemporaries and fellow countrymen of the writers, and of which some at least even now prevail among their descendants. It was quite curious to see how nasty the custom of applying pomatum to the hair could be made to appear by the exercise of a little ingenuity in the manner of describing it. In like manner nearly every doctor could point to some nursery in which the folly of the Boers is sufficiently well imitated to show that folly, at least, is not limited by conditions of either place or climate.

